# The predictive value of radiomics-based machine learning for peritoneal metastasis in gastric cancer patients: a systematic review and meta-analysis

**DOI:** 10.3389/fonc.2023.1196053

**Published:** 2023-07-03

**Authors:** Fan Zhang, Guoxue Wu, Nan Chen, Ruyue Li

**Affiliations:** Department of Pharmacy, The Fifth Clinical Medical College of Henan University of Chinese Medicine (Zhengzhou People’s Hospital), Zhengzhou, China

**Keywords:** gastric cancer, peritoneal metastasis, radiomics, machine learning, meta-analysis

## Abstract

**Background:**

For patients with gastric cancer (GC), effective preoperative identification of peritoneal metastasis (PM) remains a severe challenge in clinical practice. Regrettably, effective early identification tools are still lacking up to now. With the popularization and application of radiomics method in tumor management, some researchers try to introduce it into the early identification of PM in patients with GC. However, due to the complexity of radiomics, the value of radiomics method in the early identification of PM in GC patients remains controversial. Therefore, this systematic review was conducted to explore the feasibility of radiomics in the early identification of PM in GC patients.

**Methods:**

PubMed, Cochrane, Embase and the Web of Science were comprehensively and systematically searched up to 25 July, 2022 (CRD42022350512). The quality of the included studies was assessed using the radiomics quality score (RQS). To discuss the superiority in diagnostic accuracy of radiomics-based machine learning, a subgroup analysis was performed by machine learning (ML) based on clinical features, radiomics features, and radiomics + clinical features.

**Results:**

Finally, 11 eligible original studies covering 78 models were included in this systematic review. According to the meta-analysis, the radiomics + clinical features model had a c-index of 0.919 (95% CI: 0.871-0.969), pooled sensitivity and specificity of 0.90 (0.83-0.94) and 0.87 (0.78-0.92), respectively, in the training set, and a c- index of 0.910 (95% CI: 0.886-0.934), pooled sensitivity and specificity of 0.78 (0.71-0.84) and 0.83 (0.74-0.89), respectively, in the validation set.

**Conclusions:**

The ML methods based on radiomics + clinical features had satisfactory accuracy for the early diagnosis of PM in GC patients, and can be used as an auxiliary diagnostic tool for clinicians. However, the lack of guidelines for the proper operation of radiomics has led to the diversification of radiomics methods, which seems to limit the development of radiomics. Even so, the clinical application value of radiomics cannot be ignored. The standardization of radiomics research is required in the future for the wider application of radiomics by developing intelligent tools of radiomics.

**Systematic review registration:**

https://www.crd.york.ac.uk/PROSPERO/display_record.php?RecordID=350512, identifier CRD42022350512.

## 1 Introduction

Gastric cancer (GC) is one of the most prevalent malignant tumors and the third leading cause of cancer-related death worldwide (1, 2). Surgery is the main treatment for GC. It is of great importance for GC patients to identify peritoneal metastasis (PM) and lymph node metastasis (LNM) at an early stage before operation (3). Currently, there is no effective tool for early identification of PM in clinical practice. Radiomics was first proposed in 2003 (4) and was rapidly applied in various medical fields (5–7). It has been used in predicting chemotherapy response, prognosis and LNM in patients with GC. In some researches, radiomics was used for the early identification of PM. However, there was a significant heterogeneity due to its complexity, causing the diagnostic performance remained controversial (7–9).

Currently, the preoperative identification of PM in GC patients remains a severe challenge in clinical practice. Some researchers have explored the radiomics method for the identification of PM, but insufficient value was reported (10). With the popularization and application of radiomics in clinical practice, especially in tumor management, some researchers try to introduce it into the early identification of PM in patients with GC. However, there are diverse approaches to the implementation of radiomics due to the lack of recognized operating guidelines, and a comprehensive understanding of the predictive value of radiomics for PM in GC patients is required. Furthermore, there is still a lack of systematic understanding of the necessity of clinical variables for the implementation of radiomics in the early identification of PM in patients with GC.

Therefore, we conducted this systematic review and meta-analysis to explore the diagnostic value of ML for the early identification of PM in GC patients to provide a reference for further development of radiomics in this field.

## 2 Methods

A systematic review was conducted in strict accordance with the Preferred Reporting Items for Systematic Reviews and Meta-Analyses (PRISMA 2020), and registered on the PROSPERO platform (registration number: CRD42022350512). The registration of this systemic review is available at: https://www.crd.york.ac.uk/prospero/display_record.php?

### 2.1 Retrieval strategy

PubMed, Cochrane, Embase and Web of Science were comprehensively and systematically searched up to 25 July, 2022. Subject terms combined with free words were used. There was no restriction on region and language. The retrieval strategy is shown in the **Supplementary Material 1**.

### 2.2 Inclusion and exclusion criteria

#### 2.2.1 Inclusion criteria

1. The types of the original studies were case-control studies, cohort studies, nested case-control studies, or case-cohort studies;
2. The research subjects were GC patients;
3. The radiomics-based ML models of PM were completely constructed;
4. Studies without external validation could also be included; Currently, a large number of studies on radiomics lack external validation or independent validation sets. Even so, the contribution of these studies cannot be ignored. In our work, the results of the training set and the validation set were considered to discuss the fitting of the models, and studies without external validation or independent validation sets were included in the training set.
5. Studies of different ML models using the same dataset;
6. Studies published in English were included.

#### 2.2.2 Exclusion criteria

1. The research type was meta-analysis, review, guideline, expert opinion, etc.;
2. The complete ML model was not constructed with only differential factor analysis;
3. The following outcome measures were missing: receiver operating characteristic (ROC) curve, c-index, sensitivity, specificity, accuracy, recovery rate, precision rate, confusion matrix, diagnostic four-table, F1 score, and calibration curve, which would affect the prediction accuracy of the learning models.
4. Studies with inadequate sample sizes (<20 cases).

### 2.3 Literature screening and data extraction

The retrieved literature was imported into Endnote. After the duplicates were excluded, the original studies were initially screened by titles and abstracts. The full texts of the original studies relevant to this systematic review were downloaded, and reviewed to identify the eligible studies that were finally included in our research. Before data extraction, an extraction form was prepared for our research, including items like first author, publication year, country, patient source, stage of GC, diagnosis of PM, source of the radiomics, number of the radiography researchers, qualifications of the radiography researchers, number of all samples, number of PM samples in training set, number of samples in training set, method for generation of validation set, number of PM samples in validation set, number of samples in validation set, software for demarcating the image area, software for texture extraction, method for screening variables, types of model used, variables for modeling, evaluation of overfitting, indicators for evaluating models. Literature screening and data extraction were performed, and cross-checked by two independent investigators (GXW, RYL). Disagreements, if any, were discussed and solved with a third investigator (SLX).

### 2.4 Quality evaluation of the included studies

The radiomics quality score (RQS) was used to evaluate the quality of the included original studies by the source of radiomics data and the construction process of ML models. The RQS scale consists of 16 specific questions with a total score of 36 points. The total score of the prospective registration studies was 7 or 5 points, and were subtracted if there was no external validation (11, 12). Therefore, a large number of original studies on radiomics evaluated by the RQS scale had low scores. Two independent investigators (GXW, RYL) conducted the quality assessment and cross-checked the results. If there was any dissent, a third investigator (SLX) was consulted to assist in adjudication.

### 2.5 Outcome measures

The primary outcome measure of this systematic review was the c-index which reflected the accuracy of the model. However, the c-index could not truly reflect the accuracy of the ML model on PM if there was a serious imbalance between the number of PM samples and non-PM samples. Therefore, sensitivity and specificity were also used as outcome measures to overcome this shortcoming.

### 2.6 Statistical analysis

A meta-analysis of the measures (c-index and accuracy) was performed to evaluate ML models. If 95% confidence interval and standard error of the c-index were missed out, the study by Debray TP et al. (13) was used as a reference to estimate the standard error. If there was a lack of accuracy in the original study, the accuracy was calculated based on sensitivity, specificity, the number of samples of each molecular subtype and the number of samples used in modeling. Considering the differences in the variables included in the learning models and in the parameters, a random effects model was preferred in this meta-analysis. R4.2.0 (R development Core Team, Vienna, http://www.R-project.org) was used for this meta-analysis.

## 3 Results

### 3.1 Literature retrieval

**Figure 1:**
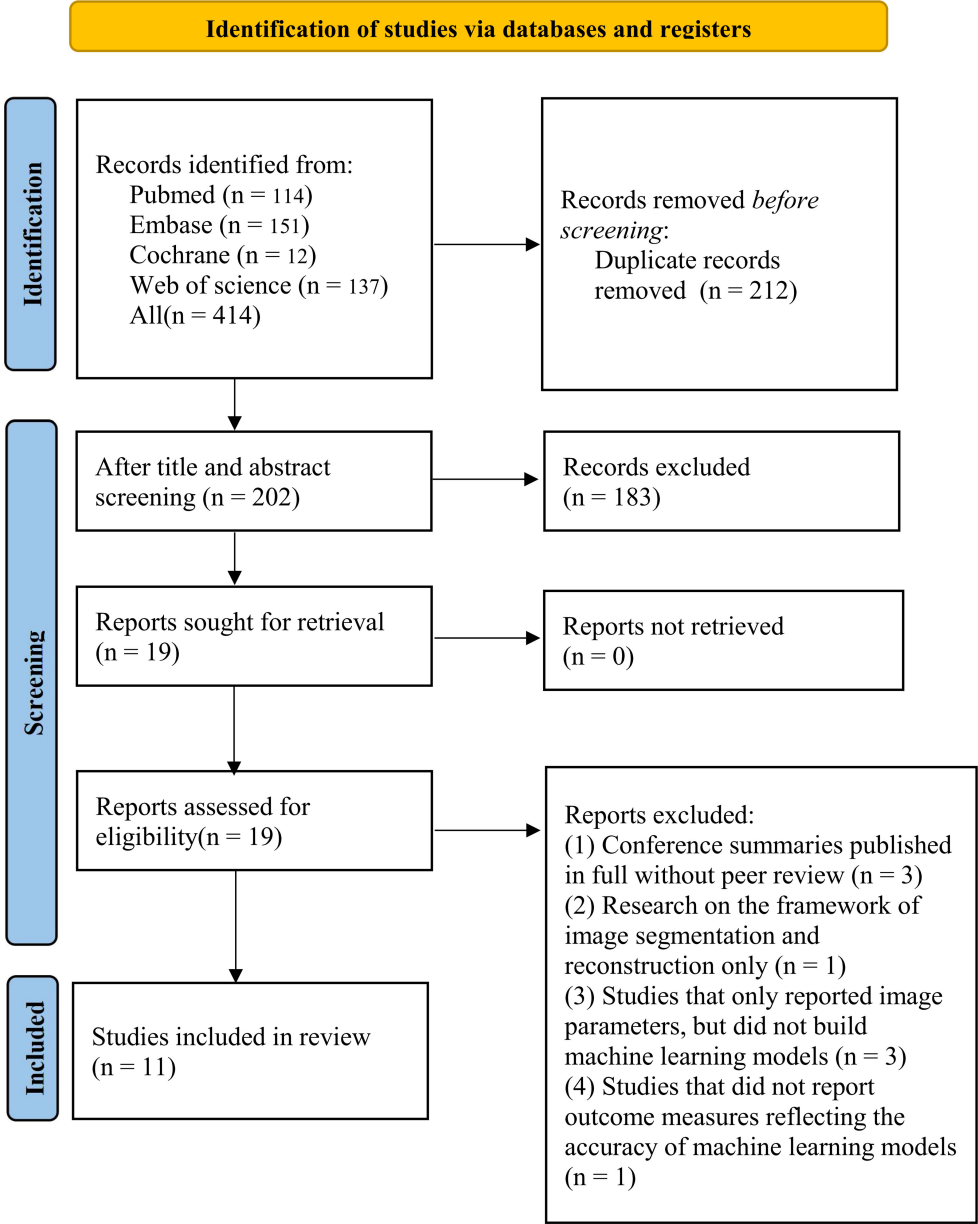
PRISMA diagram (PRISMA 2020).

A total of 414 studies were initially identified, of which 212 were duplicated studies, 183 were excluded by reviewing the titles and abstracts, including studies irrelevant to our topic, reviews, letters or comments, and non-English studies. The full texts of the remaining 19 studies were downloaded, and the following studies were excluded, including 3 published conference abstracts with the full texts without peer review, one image segmentation study, and one published conference abstract without full texts and peer review. Finally, 11 studies were included (**Figure 1**).

### 3.2 Basic characteristics of the studies

The 11 included studies were mainly published from 2019 to 2022 (one in 2019, two in 2020, five in 2021, and three in 2022) as shown in **Table 1**. The radiomics data were from CT/PET/DECT. There were 33 models in the training set and 45 models in the validation set. In the training set model, six were constructed using clinical features alone, 12 were constructed using radiomics features alone, and seven were constructed using radiomics + clinical features. In the validation set, 15 were constructed using clinical features alone, 17 were constructed using radiomic alone, and 11 were constructed using radiomics + clinical features. There were six single-centre studies and five multi-centre studies.

**Table 1:** Characteristics of included studies.

No.	Author	Year	Country	Patient source	Number of peritoneal metastasis samples in training set		Number of samples in training set		Method for the generation of validation set	Number of peritoneal metastasis samples in validation set	Number of samples in validation set
1	D Dong	2019	China	four centres	50		100		other centres	20 27 24	226 131 97
2	Seyedehnafiseh.M	2021	USA	single-centre	121		159		cross validation		
3	Weicai Huang	2020	China	two centres	90		562		other centres	17 62	106 287
4	Lili Wang	2022	China	two centres	30		393		other centres	19 18	215 202
5	Shunli Liu	2020	China	single-centre	34		158		random sampling	11	75
6	Jiang Huang	2022	China	single-centre	49		98		according to the date of CT examination	15	30
7	Beihui Xue	2021	China	single-centre	77		250		random sampling	32	105
8	Yong Chen	2021	China	single-centre	43		160		random sampling	22	79
9	Yuming Jiang	2021	USA	two centres	135		1225		other centres	138 32	504 297
10	Dan Liu	2021	China	three centres	58		395		other centres	21 14	149 55
11	Giorgio Maria Masci	2022	Italy	single-centre	45		90		NA	NA	NA

No.	Author	Year	The source of the radiomics	Software for demarcating the image area			Method for screening variables		Types of the model	Variables for modeling	
1	D Dong	2019	CT	ITK-SNAP			LASSO		LR	radiomics + clinical features	
2	Seyedehnafiseh.M	2021	CT				Random projection		GBM/LR/SVM/RF/DT	radiomic	
3	Weicai Huang	2020	CT	ITK-SNAP			LASSO		LR	clinical features radiomic radiomics + clinical features	
4	Lili Wang	2022	CT	MaZda 4.6			LASSO		NB/LR	clinical features radiomic radiomics + clinical features	
5	Shunli Liu	2020	CT				LASSO		CV/LR/SVM	clinical features radiomic radiomics + clinical features	
6	Jiang Huang	2022	CT	RadCloud			LASSO		SVM/LR/MLP/RF	clinical features radiomic radiomics + clinical features	
7	Beihui Xue	2021	PET/CT	LIFEX			LASSO		LR	clinical features radiomic radiomics + clinical features	
8	Yong Chen	2021	DECT	LIFEx			Boruta		DL/RF	clinical features radiomic radiomics + clinical features	
9	Yuming Jiang	2021	CT	ITK-SNAP					DL	clinical pathology	
10	Dan Liu	2021	CT	ITK-SNAP							
11	Giorgio Maria Masci	2022	Italy	LIFEx					LR	radiomic	

NA indicated that no relevant information was provided in the original study.

### 3.3 Quality assessment

Among the 11 included studies, diagnostic protocols were fully described in five studies (45%). However, none of the studies used public protocols. Nine studies (81%) clearly described the demarcation of imaging areas involving two or more clinicians. None of the studies performed pre-experiments to adjust the equipment under different parameters before the experiments. One study analyzed the robustness by time variation. One study analyzed the cutoff values. Five (45%) studies conducted external validation. Eleven (100%) studies indicated that their prediction models had better performance compared with the current “gold standard” in determining PM in patients with GC. One study publicly reported the code and data. None of the included studies were prospectively validated. Finally, the lowest comprehensive score was 2.78% and the highest was 52.78%. The detailed final RQS scores are shown in **Table 2**.

**Table 2:** Quality evaluation table.

Subject	Author	Year	V1	V2	V3	V4	V5	V6	V7	V8	V9	V10	V11	V12	V13	V14	V15	V16
Development and validation of an individualized nomogram to identify occult peritoneal metastasis in patients with advanced gastric cancer	D Dong	2019	0	0	0	0	0	1	0	0	2	2	0	4	2	2	0	0
Applying a random projection algorithm to optimize machine learning model for predicting peritoneal metastasis in gastric cancer patients using CT images	Seyedehnafiseh Mirniaharikandehei	2021	1	1	0	1	0	1	0	0	2	2	0	2	2	2	0	0
Radiomics Nomogram for Prediction of Peritoneal Metastasis in Patients With Gastric Cancer	Weicai Huang	2020	1	1	0	0	0	1	0	0	2	2	0	3	2	2	0	0
Novel CT based clinical nomogram comparable to radiomics model for identification of occult peritoneal metastasis in advanced gastric cancer	Lili Wang	2022	1	1	0	0	0	1	0	0	1	1	0	3	2	2	0	0
Radiomics analysis using contrast-enhanced CT for preoperative prediction of occult peritoneal metastasis in advanced gastric cancer	Shunli Liu	2020	0	1	0	0	0	1	0	1	2	2	0	2	2	0	0	0
Comparison of clinical-computed tomography model with 2D and 3D radiomics models to predict occult peritoneal metastases in advanced gastric cancer	Jiang Huang	2022	0	1	0	0	0	1	0	0	1	1	0	2	2	0	0	0
Role of CT texture analysis for predicting peritoneal metastases in patients with gastric cancer	Giorgio Maria Masci	2022	0	1	0	0	0	1	0	0	1	1	0	-5	2	0	0	0
Development and Validation of a Radiomics Model Based on F-18-FDG PET of Primary Gastric Cancer for Predicting Peritoneal Metastasis	Beihui Xue	2021	0	1	0	0	0	1	0	0	1	1	9	2	2	2	0	0
Dual-Energy Computed Tomography-Based Radiomics to Predict Peritoneal Metastasis in Gastric Cancer	Yong Chen	2021	1	1	0	0	0	1	0	0	2	2	0	2	2	2	0	0
Noninvasive Prediction of Occult Peritoneal Metastasis in Gastric Cancer Using Deep Learning	Yuming Jiang	2021	1	0	0	0	0	1	0	0	2	2	0	4	2	2	0	1
A Bounding Box-Based Radiomics Model for Detecting Occult Peritoneal Metastasis in Advanced Gastric Cancer: A Multicenter Study	Dan Liu	2021	0	1	0	0	0	1	0	0	2	2	0	3	2	1	0	0

### 3.4 Meta-analysis

#### 3.4.1 C-index

**Figure 2:**
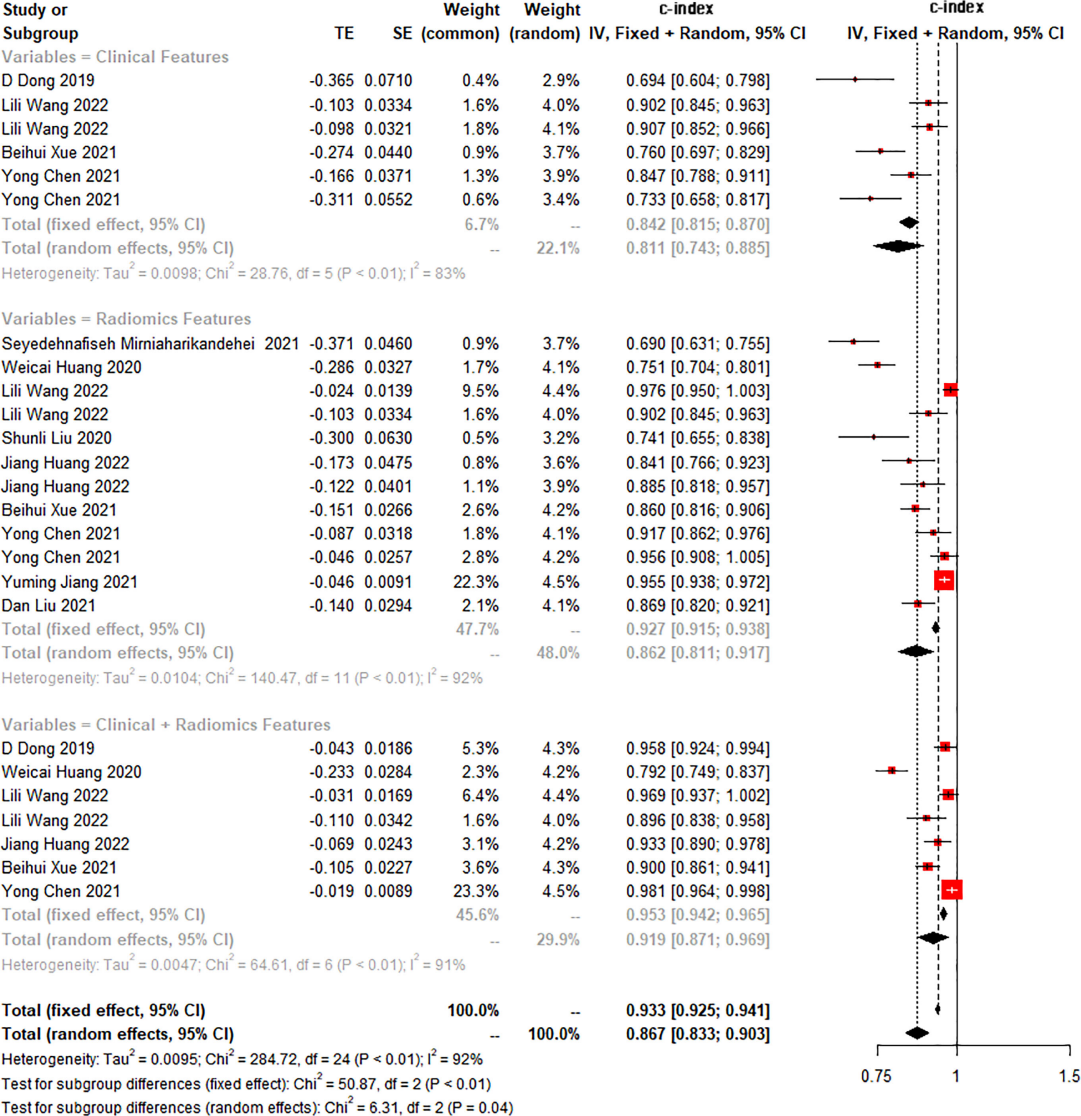
Forest plot of meta-analysis of c-index of machine learning for identifying peritoneal metastasis in gastric cancer patients in the training set.

**Figure 3:**
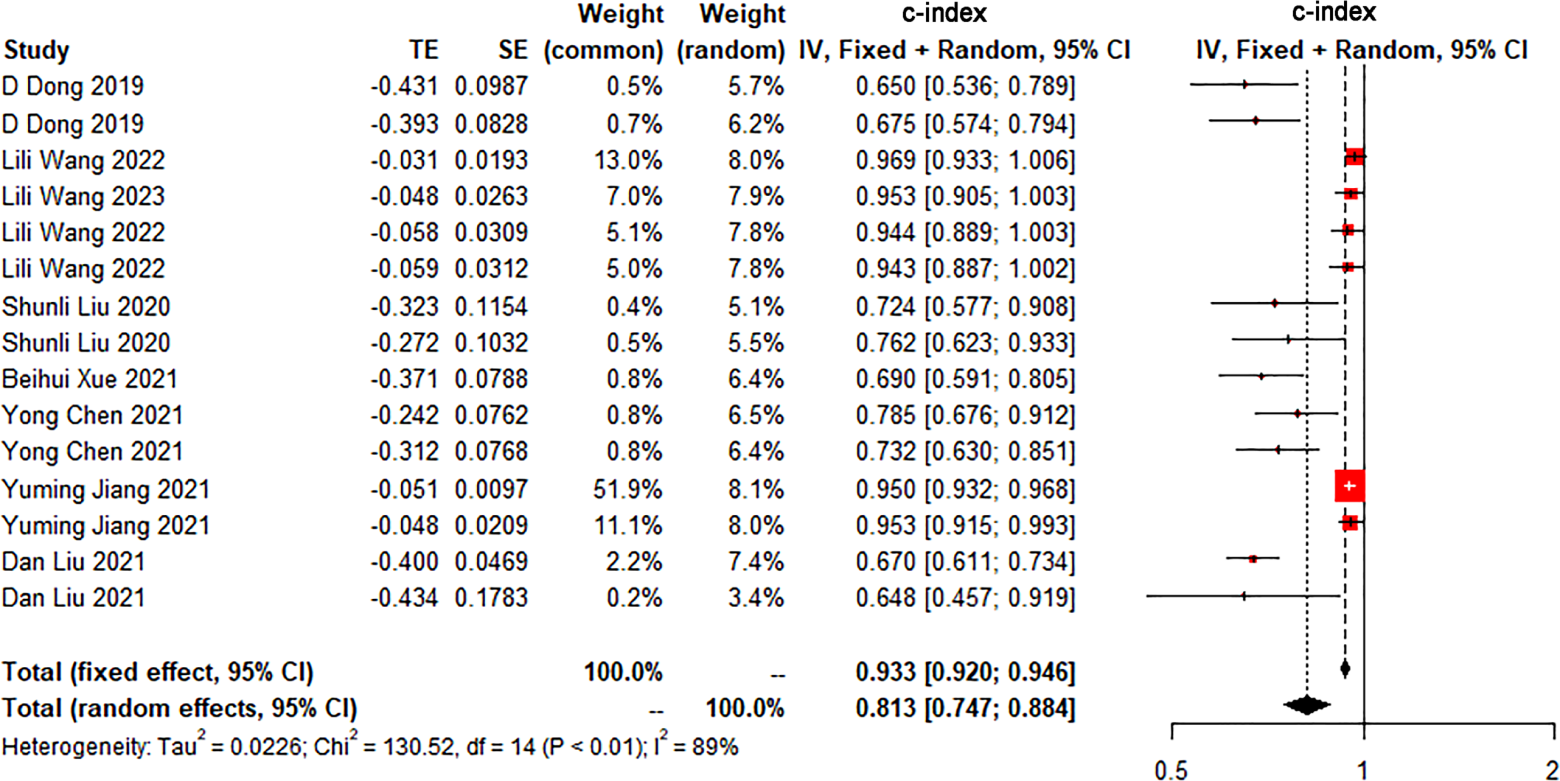
Forest plot of meta-analysis of c-index of machine learning based on clinical features for identifying peritoneal metastasis in gastric cancer patients in the validation set.

**Figure 4:**
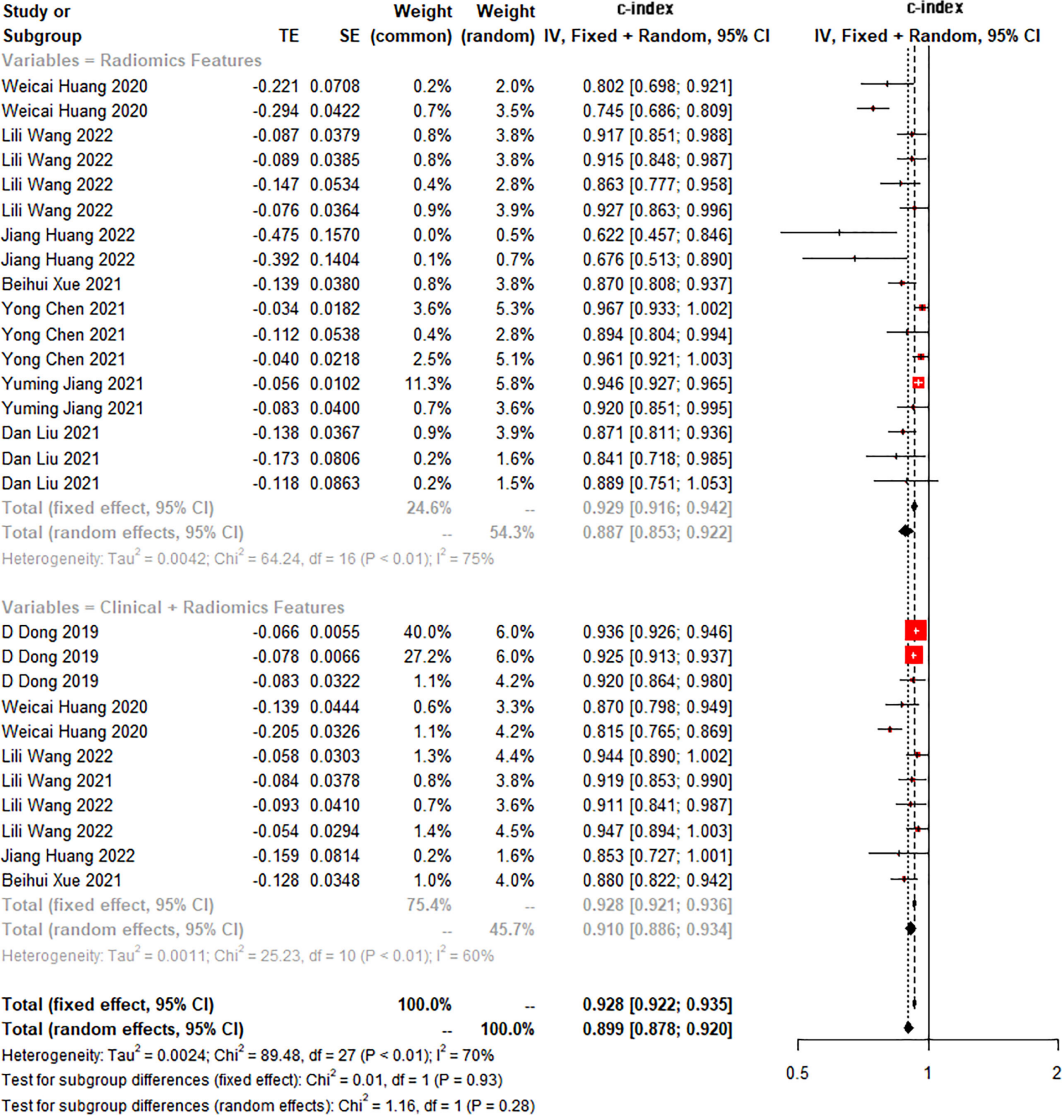
Forest plot of meta-analysis of c-index of machine learning based on radiomics and clinical features for identifying peritoneal metastasis in gastric cancer patients in the validation set.

The c-index is a common indicator for evaluating the overall accuracy of a model. Usually, the results of an independent validation set can better reflect the true accuracy of the model. However, for ML, overfitting or underfitting may occur in the modeling process. Therefore, it is necessary to evaluate the c-index of the ML model in the training set and the validation set. In the training cohort and independent validation cohort, radiomics-based ML showed a higher c-index than ML constructed based on clinical features. ML based on radiomics + clinical features showed the best performance, with a c-index of 0.919 (95%CI: 0.871-0.969) and 0.910 (95%CI: 0.886-0.934) in the training set and the verification set, respectively (**Figures 2**–**4** and **Table 3**).

**Table 3:** Meta-analysis of c-index of radiomics-based ML.

Modeling variables	Training set			Validation set		
	Number	c-index (95%CI)	I2 (%)	Number	c-index (95%CI)	I2 (%)
Clinical features	6	0.811 (0.743-0.885)	83	15	0.813 (0.747-0.884)	89
Radiomics	12	0.862 (0.811-0.917)	92	17	0.887 (0.853-0.922)	75
Radiomics + clinical features	7	0.919 (0.871-0.969)	91	11	0.910 (0.886-0.934)	70

Number in the training set represents the number of models, Number in the validation set represents the number of cohorts for model verification, and I^2^ represents the heterogeneity index.

We found that the c-index of the ML model constructed based on clinical features, radiomics, and radiomics + clinical features was very similar in the training set and validation set, indicating that there was no overfitting in the modeling process (**Table 3**). Furthermore, no significant publication bias for c-index was found (**Supplementary Material 2**).

#### 3.4.2 Sensitivity and specificity

In both the training and validation cohorts, the ML models based on clinical features showed unsatisfactory sensitivity but good specificity, indicating that clinical features can ideally help identify patients without PM but have a limited ability to identify patients with PM. Compared with ML models constructed by clinical features, radiomics-based ML models had significant higher sensitivity, but the specificity was not significantly improved in the validation set. Overall, ML based on radiomics+clinical features has favorable sensitivity and specificity. The sensitivity and specificity are 0.90 (95%CI:0.83-0.94) and 0.87 (95%CI:0.78 -0.92) in the training set, and 0.78 (95%CI:0.70-0.85) and 0.90 (95%CI:0.86-0.93) in the validation set, respectively (**Supplementary Material 3** and **Table 4**).

**Table 4:** Meta-analysis of sensitivity and specificity of radiomics-based ML.

Modeling variables	Training set			Validation set		
	Number	Sen (95%CI)	Spe (95%CI)	Number	Sen (95%CI)	Spe (95%CI)
Clinical features	6	0.63 (0.42-0.80)	0.91 (0.81-0.96)	15	0.53 (0.32-0.73)	0.81 (0.60-0.93)
Radiomics	12	0.76 (0.69-0.82)	0.79 (0.66-0.88)	17	0.79 (0.70-0.86)	0.89 (0.80-0.94)
Radiomics + clinical features	7	0.90 (0.83-0.94)	0.87 (0.78-0.92)	11	0.78 (0.70-0.85)	0.90 (0.86-0.93)

Number in the training set represents the number of models; Number in the validation set represents the number of cohorts for model verification; Sen represents sensitivity, and Spe represents specificity.

## 4 Discussion

A total of 11 original studies were included in this systematic review. It has been demonstrated that ML, especially radiomics-based ML, is an ideal method for predicting and identifying PM in GC patients before operation. At the same time, Ml based only on clinical features should not be ignored. Therefore, as was shown this study, Ml constructed based on both clinical features and radiomic is the most effective for predicting PM in GC patients before operation.

The incidence of PM in GC patients cannot be neglected in clinical practice (14). However, early identification of PM in patients with GC is highly challenging. Despite considerable efforts to explore various detection methods, an ideal early non-invasive detection method has not been found. A systematic review by Zhen Wang (10) explored the efficiency of ultrasonography(US), Endoscopic ultrasound(EUS), computed tomography (CT), magnetic resonance imaging(MRI), and 18F-fluorodeoxyglucose positron emission tomography (18F-FDG PET) in the early detection of PM in GC patients. EUS showed the highest sensitivity [34% (95%CI: 10% - 69%)), followed by CT (33% (95%CI: 16% - 56%)], while each method had a very high specificity (all >96%). The systematic review by Wang et al. (15) included 5 original studies, revealing that [68Ga] Ga-FAPI-04 PET MRI/CT outperformed [18F]-FDG PET MRI/CT in the detection of PM in patients with GC according to their sensitivity. However, the sensitivity and specificity were not described in detail in their study, which made it difficult for people to understand the application value of [68Ga] Ga-FAPI-04 PET MRI/CT in the detection of PM in GC patients. In addition, the systematic review by I van ‘t Sant et al. (16) showed that CT, PET/CT, and MRI had ideal sensitivity and specificity; however, patients with gastrointestinal tumors and ovarian cancer were not distinguished in their study. It seems indicate that radiographic images alone are relatively accurate in the diagnosis of PM in other tumors, but have limited performance in the diagnostic of PM in GC. R F Ramos et al (17) have found based on limited evidence that laparoscopic staging of GC has an ideal sensitivity (84.6%) for the detection of PM, but it is invasive. Our systematic review shows that radiomics-based methods have ideal sensitivity and specificity for detecting PM in GC patients, and therefore, can be used as an adjunct method for early diagnosis.

Even though ML based on radiomics and clinical features is shown to have an ideal value for the early detection of PM in GC patients, there are some challenges in the systematic review of radiomics. At present, the methods of radiomics are diverse. In terms of the demarcation of radiomics, its diversity is a major source of heterogeneity. First, the plotting of the region of interest (ROI) is highly subjective. Demarcation is often conducted by senior clinicians, and there is no uniform standard. Second, although software is mostly used to extract texture features, there may be differences in the parameters. Third, the types of ML methods used in modeling and the methods for feature extraction are different, and there is no unified standard. The main methods for feature extraction are mostly LASSO regression or univariate analysis, which causes significant bias. The selection criteria for ML methods are also different. It is impossible to use a general standard for the application of radiomics in various fields. Hence, the development of radiomics-based ML lacks a recognized operating guideline and is limited by researchers’ experience in the original research process, resulting in the existence of diversified methods. The diversity may be attributable to the differences in the selection of radiomics sources, ROI division, texture extraction, variable screening or dimensionality reduction, model construction and validation. Therefore, it is a great challenge for radiomics to be universally applied in clinical practice.

The diversified methods of radiomics may cause high heterogeneity, but the application value of radiomics methods is undeniable in clinical practice in recent years. Radiomics shows a high value in the overall risk management of GC (18, 19). Yuming Jiang et al. (20) used advanced deep learning techniques to improve the prediction of GC recurrence, which showed higher performance in prognostic prediction than the currently applied TNM staging system. A nomogram constructed by Wenjuan Zhang et al. (21) based on clinical risk factors for early recurrence of GC demonstrated potent prognostic effects in both the training set and testing set, with a c-index of 0.831 (95% CI, 0.786 - 0.876) and 0.826 (95% CI, 0772 - 0.880), respectively. Zelan Ma et al. (22) developed a CT-based pre-treatment radiomic signature that could effectively distinguish Borrmann type IV GC from primary gastric lymphoma. Qinmei Xu et al. (23) proved that a CT-based radiomics model had a predictive value for advanced gastric cancer before, during, and at the end of neoadjuvant chemotherapy. Lili Wang et al. (24) reported that CT-based radiomics could effectively predict No.10 lymph node (LNs) metastasis in patients with advanced proximal gastric cancer (APGC) before operation. Xujie Gao et al. (25) proposed a radiomics model based on radiomics features and CT reports as a noninvasive approach for estimating lymph node metastasis in GC at an early stage before operation. This model showed satisfactory discriminative performance in the training cohort (c-index = 0.91) and testing cohort (c-index = 0.89). Yue Wang et al. (26) constructed a radiomics nomogram which could successfully identify lymph node metastasis in GC. This nomogram showed satisfactory performance in both training and testing cohorts, with the c-index of 0.886 (95% CI, 0.808 to 0.941) and 0.881 (95% CI, 0.759 to 0.956), respectively. Jing Li et al. (27) constructed a DECT-based deep ML model for detecting lymph node metastasis in FC, with the c-index of 0.839 (95% CI, 0.773 to 0.904) in the training set and 0.821 (95% CI, 0722 to 0.920) in the test set. This model was superior to the single-energy model and clinical model. Our study showed that the model based on radiomics + clinical features had significant value in predicting PM in GC before operation, providing novel insights into the identification of PM before operation.

This study was the first systematic review investigating and complementing the application of radiomics in the detection of PM in GC. Furthermore, our study demonstrated the feasibility of predicting peritoneal cancer in GC patients based on radiomics. However, there were also some limitations in our study. First, although a comprehensive and systematic search was performed, the number of included studies was relatively small. Second, there were various methods of ML included in this study. Due to the limited numbers of included studies, t the differences between various ML could not be investigated under different modeling variables.

### 4.1 Suggestions for future work

Existing original research on radiomics has several limitations, which should be improved in the future implementation. First, in the acquisition of imaging data, the influence of correcting over-configuration of imaging equipment is not considered. Second, the extraction process of texture features is not reported. Third, overfitting or underfitting in the modeling process is not considered. Fourth, multi-center verification is rarely performed in the original studies.

## 5 Conclusion

Despite the favorable predictive value of radiomics-based ML for the PM in GC patients, ML models based on clinical features or radiomics + clinical features are highly accurate in the early diagnosis of PM in GC patients and can be used as an auxiliary diagnostic tool for clinicians. However, guidelines for the proper operation of radiomics are lacking, leading to the diversity in radiomics methods and thus limiting the development of radiomics. Even in this context, the clinical application value of radiomics should not be ignored. Therefore, standardized radiomics research is warranted to develop radiomics-based intelligent tools with wider application.

## Data availability statement

The original contributions presented in the study are included in the article/**Supplementary Material**. Further inquiries can be directed to the corresponding author.

## Author contributions

FZ wrote the main manuscript and fully participated in all analyses. GW and RL participated in literature search, data extraction, and quality assessment. NC contributed to the study concept and design. All authors contributed to the article and approved the submitted version.
