# Supplementary material for: The predictive value of radiomics-based machine learning for peritoneal metastasis in gastric cancer patients: a systematic review and meta-analysis
Source: Front Oncol. 2023 Jul 3;13:1196053. doi: 10.3389/fonc.2023.1196053 (PMC10352083; doi:10.3389/fonc.2023.1196053)
Supplement: Supplementary file 1 [file DataSheet_1.docx]

Supplementary Material

The predictive value of radiomics - based machine learning for peritoneal metastasis in gastric cancer patients: a systematic review and meta-analysis

Fan Zhang^*^, Guoxue Wu, Nan Chen, Ruyue Li

*** Correspondence:** Fan Zhang: 15803824009@163.com

# **Supplementary Material 1:** Literature Search Strategy.

**1.Pubmed**

| Search number | Query | Results |
| --- | --- | --- |
| #1 | "Stomach Neoplasms""[Mesh]" | 106,458 |
| #2 | ((((((((((((((((((((((((((((((((((((Stomach Neoplasms[Title/Abstract]) OR (Neoplasm, Stomach[Title/Abstract])) OR (Stomach Neoplasm[Title/Abstract])) OR (Neoplasms, Stomach[Title/Abstract])) OR (Gastric Neoplasms[Title/Abstract])) OR (Gastric Neoplasm[Title/Abstract])) OR (Neoplasm, Gastric[Title/Abstract])) OR (Neoplasms, Gastric[Title/Abstract])) OR (Cancer of Stomach[Title/Abstract])) OR (Stomach Cancers[Title/Abstract])) OR (Gastric Cancer[Title/Abstract])) OR (Cancer, Gastric[Title/Abstract])) OR (Cancers, Gastric[Title/Abstract])) OR (Gastric Cancers[Title/Abstract])) OR (Stomach Cancer[Title/Abstract])) OR (Cancer, Stomach[Title/Abstract])) OR (Cancers, Stomach[Title/Abstract])) OR (Cancer of the Stomach[Title/Abstract])) OR (Gastric Cancer, Familial Diffuse[Title/Abstract])) OR (stomach cancer[Title/Abstract])) OR (stomach tumor[Title/Abstract])) OR (gastric tumor[Title/Abstract])) OR (gastric tumour[Title/Abstract])) OR (mucosa tumor, stomach[Title/Abstract])) OR (mucosa tumour, stomach[Title/Abstract])) OR (stomach mucosa tumor[Title/Abstract])) OR (stomach mucosa tumour[Title/Abstract])) OR (stomach neoplasia[Title/Abstract])) OR (stomach neoplasm[Title/Abstract])) OR (stomach neoplasms[Title/Abstract])) OR (stomach tumour[Title/Abstract])) OR (stomach ulcerated tumor[Title/Abstract])) OR (stomach ulcerated tumour[Title/Abstract])) OR (stomach ulcerating tumor[Title/Abstract])) OR (stomach ulcerating tumour[Title/Abstract])) OR (tumor, stomach mucosa[Title/Abstract])) OR (tumour, stomach mucosa[Title/Abstract]) | 93,721 |
| #3 | #1 OR #2 | 132,915 |
| #4 | ((((Radiomics[Title/Abstract]) OR (radiomic[Title/Abstract])) OR (radiogenomic[Title/Abstract])) OR (radiomics-based[Title/Abstract])) OR (radiomic signature[Title/Abstract]) | 6,317 |
| #5 | #3 AND #4 | 114 |

**2.Cochrane**

| Search number | Query | Results |
| --- | --- | --- |
| #1 | MeSH descriptor: [Stomach Neoplasms] explode all trees | 2889 |
| #2 | (Stomach Neoplasms):ti,ab,kw OR (Neoplasm, Stomach):ti,ab,kw OR (Stomach Neoplasm):ti,ab,kw OR (Neoplasms, Stomach):ti,ab,kw OR (Gastric Neoplasms):ti,ab,kw | 4395 |
| #3 | (Gastric Neoplasm):ti,ab,kw OR (Neoplasm, Gastric):ti,ab,kw OR (Neoplasms, Gastric):ti,ab,kw OR (Cancer of Stomach):ti,ab,kw OR (Stomach Cancers):ti,ab,kw | 7611 |
| #4 | (Gastric Cancer):ti,ab,kw OR (Cancer, Gastric):ti,ab,kw OR (Cancers, Gastric):ti,ab,kw OR (Gastric Cancers):ti,ab,kw OR (Stomach Cancer):ti,ab,kw | 9547 |
| #5 | (Cancer, Stomach):ti,ab,kw OR (Cancers, Stomach):ti,ab,kw OR (Cancer of the Stomach):ti,ab,kw OR (Gastric Cancer, Familial Diffuse):ti,ab,kw OR (stomach cancer):ti,ab,kw | 6700 |
| #6 | (stomach tumor):ti,ab,kw OR (gastric tumor):ti,ab,kw OR (gastric tumour):ti,ab,kw OR (mucosa tumor, stomach):ti,ab,kw OR (mucosa tumour, stomach):ti,ab,kw | 3139 |
| #7 | (stomach mucosa tumor):ti,ab,kw OR (stomach mucosa tumour):ti,ab,kw OR (stomach neoplasia):ti,ab,kw OR (stomach neoplasm):ti,ab,kw OR (stomach neoplasms):ti,ab,kw | 4005 |
| #8 | (stomach tumour):ti,ab,kw OR (stomach ulcerated tumor):ti,ab,kw OR (stomach ulcerated tumour):ti,ab,kw OR (stomach ulcerating tumor):ti,ab,kw OR (stomach ulcerating tumour):ti,ab,kw | 2306 |
| #9 | (tumor, stomach mucosa):ti,ab,kw OR (tumour, stomach mucosa):ti,ab,kw | 135 |
| #10 | #1 OR #2 OR #3 OR #4 OR #5 OR #6 OR #7 OR #8 OR #9 | 10848 |
| #11 | (Radiomics):ti,ab,kw OR (radiomic):ti,ab,kw OR (radiogenomic):ti,ab,kw OR (radiomics-based):ti,ab,kw OR (radiomic signature):ti,ab,kw | 436 |
| #12 | #10 and #11 | 12 |

**3.Embase**

| Search number | Query | Results |
| --- | --- | --- |
| #1 | 'stomach tumor '/exp | 184653 |
| #2 | "'neoplasm, stomach':ab,ti OR 'neoplasms, stomach':ab,ti OR 'gastric neoplasms':ab,ti OR 'gastric neoplasm':ab,ti OR 'neoplasm, gastric':ab,ti OR 'neoplasms, gastric':ab,ti OR 'cancer of stomach':ab,ti OR 'stomach cancers':ab,ti OR 'gastric cancer':ab,ti OR 'cancer, gastric':ab,ti OR 'cancers, gastric':ab,ti OR 'gastric cancers':ab,ti OR 'cancer, stomach':ab,ti OR 'cancers, stomach':ab,ti OR 'cancer of the stomach':ab,ti OR 'gastric cancer, familial diffuse':ab,ti OR 'stomach cancer':ab,ti OR 'gastric tumor':ab,ti OR 'gastric tumour':ab,ti OR 'mucosa tumor, stomach':ab,ti OR 'mucosa tumour, stomach':ab,ti OR 'stomach mucosa tumor':ab,ti OR 'stomach mucosa tumour':ab,ti OR 'stomach neoplasia':ab,ti OR 'stomach neoplasm':ab,ti OR 'stomach neoplasms':ab,ti OR 'stomach tumour':ab,ti OR 'stomach ulcerated tumor':ab,ti OR 'stomach ulcerated tumour':ab,ti OR 'stomach ulcerating tumor':ab,ti OR 'stomach ulcerating tumour':ab,ti OR 'tumor, stomach mucosa':ab,ti OR 'tumour, stomach mucosa':ab,ti" | 113719 |
| #3 | #1 OR #2 | 197426 |
| #4 | 'radiomics'/exp | 5197 |
| #5 | "radiomics:ab,ti OR radiomic:ab,ti OR radiogenomic:ab,ti OR 'radiomics based':ab,ti OR 'radiomic signature':ab,ti" | 8608 |
| #6 | #4 OR #5 | 9099 |
| #7 | #3 AND #6 | 151 |

**4.Web of science**

| Search number | Query | Results |
| --- | --- | --- |
| #1 | Stomach Neoplasms (Topic) or Neoplasm, Stomach (Topic) or Stomach Neoplasm (Topic) or Neoplasms, Stomach (Topic) or Gastric Neoplasms (Topic) or Gastric Neoplasm (Topic) or Neoplasm, Gastric (Topic) or Neoplasms, Gastric (Topic) or Cancer of Stomach (Topic) or Stomach Cancers (Topic) or Gastric Cancer (Topic) or Cancer, Gastric (Topic) or Cancers, Gastric (Topic) or Gastric Cancers (Topic) or Stomach Cancer (Topic) or Cancer, Stomach (Topic) or Cancers, Stomach (Topic) or Cancer of the Stomach (Topic) or Gastric Cancer, Familial Diffuse (Topic) or stomach cancer (Topic) or stomach tumor (Topic) or gastric tumor (Topic) or gastric tumour (Topic) or mucosa tumour, stomach (Topic) or mucosa tumor, stomach (Topic) or stomach mucosa tumor (Topic) or stomach mucosa tumour (Topic) or stomach neoplasia (Topic) or stomach neoplasm (Topic) or stomach neoplasms (Topic) or stomach tumour (Topic) or stomach ulcerated tumor (Topic) or stomach ulcerated tumour (Topic) or stomach ulcerating tumor (Topic) or stomach ulcerating tumour (Topic) or tumor, stomach mucosa (Topic) or tumour, stomach mucosa (Topic) | 146,135 |
| #2 | Radiomics (Topic) or radiomic (Topic) or radiogenomic (Topic) or radiomics-based (Topic) or radiomic signature (Topic) | 8.892 |
| #3 | #2 AND #1 | 137 |
