# Supplementary material for: The predictive value of radiomics-based machine learning for peritoneal metastasis in gastric cancer patients: a systematic review and meta-analysis
Source: Front Oncol. 2023 Jul 3;13:1196053. doi: 10.3389/fonc.2023.1196053 (PMC10352083; doi:10.3389/fonc.2023.1196053)
Supplement: Supplementary file 2 [file DataSheet_2.docx]

Supplementary Material

The predictive value of radiomics - based machine learning for peritoneal metastasis in gastric cancer patients: a systematic review and meta-analysis

Fan Zhang^*^, Guoxue Wu, Nan Chen, Ruyue Li

*** Correspondence:** Fan Zhang: 15803824009@163.com

# Supplementary Material 2: Funnel plot of meta-analysis of sensitivity and specificity of machine learning based on clinical features, radiomics features, and radiomics+clinical features for identifying peritoneal metastasis in gastric cancer patients.

**Table of contents**

Fig. S1 Funnel plot for machine learning of clinical features in the training set

Fig. S2 Funnel plot of radiomics machine learning for training set

Fig. S3 Funnel plot for training set radiomics joint clinical feature machine learning


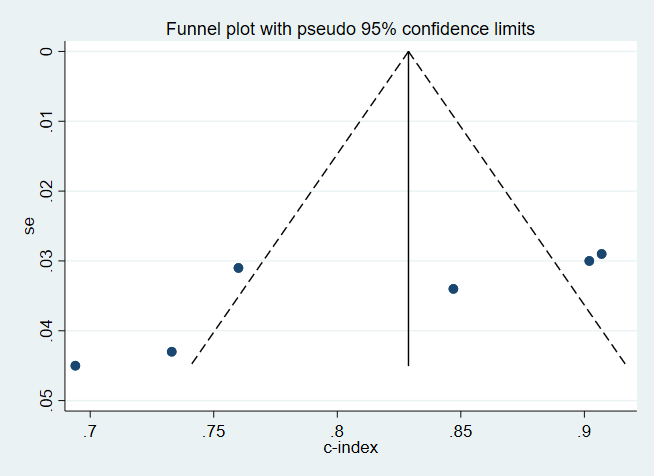


Fig. S1 Funnel plot for machine learning of clinical features in the training set


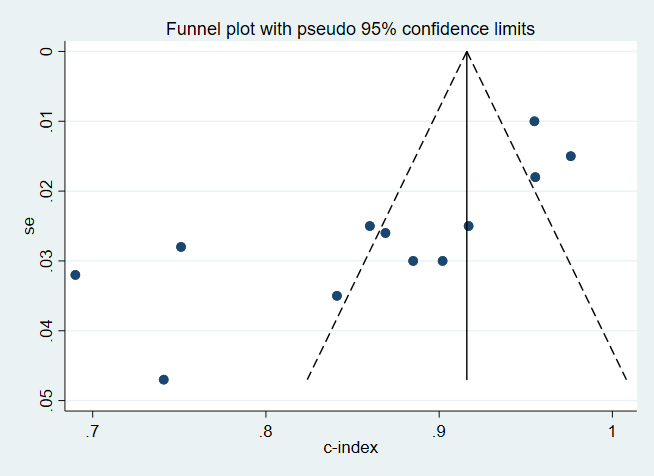


Fig. S2 Funnel plot of radiomics machine learning for training set


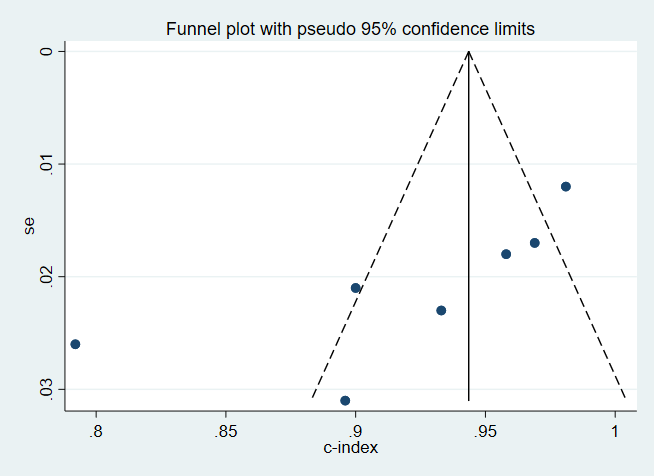


Fig. S3 Funnel plot for training set radiomics joint clinical feature machine learning
