# Supplementary material for: The predictive value of radiomics-based machine learning for peritoneal metastasis in gastric cancer patients: a systematic review and meta-analysis
Source: Front Oncol. 2023 Jul 3;13:1196053. doi: 10.3389/fonc.2023.1196053 (PMC10352083; doi:10.3389/fonc.2023.1196053)
Supplement: Supplementary file 3 [file DataSheet_3.docx]

Supplementary Material

The predictive value of radiomics - based machine learning for peritoneal metastasis in gastric cancer patients: a systematic review and meta-analysis

Fan Zhang^*^, Guoxue Wu, Nan Chen, Ruyue Li

*** Correspondence:** Fan Zhang: 15803824009@163.com

# Supplementary Material 3: The forest plot of sensitivity and specificity for predicting peritoneal metastasis in gastric cancer.

**Table of contents**

Fig. S1 Machine learning models in the training set

Fig. S2 Radiomics-based machine learning models in the training set

Fig. S3 Clinical features-based machine learning models in the training set

Fig. S4 Machine learning models based on both clinical features and radiomics in the training set

Fig. S5 Machine learning models in the validation set

Fig. S6 Radiomics-based machine learning models in the validation set

Fig. S7 Clinical features-based machine learning models in the validation set

Fig. S8 Machine learning models based on both clinical features and radiomics in the validation set


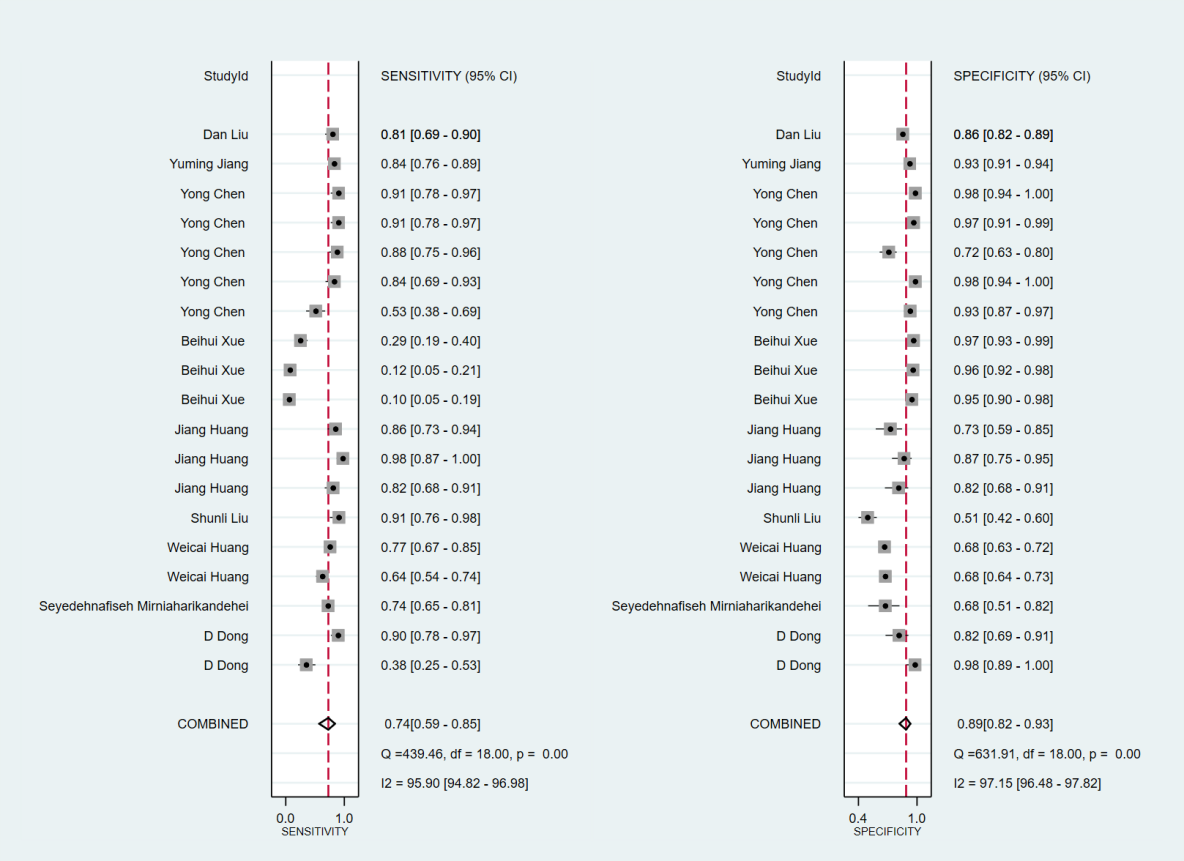


Fig. S1 Machine learning models in the training set


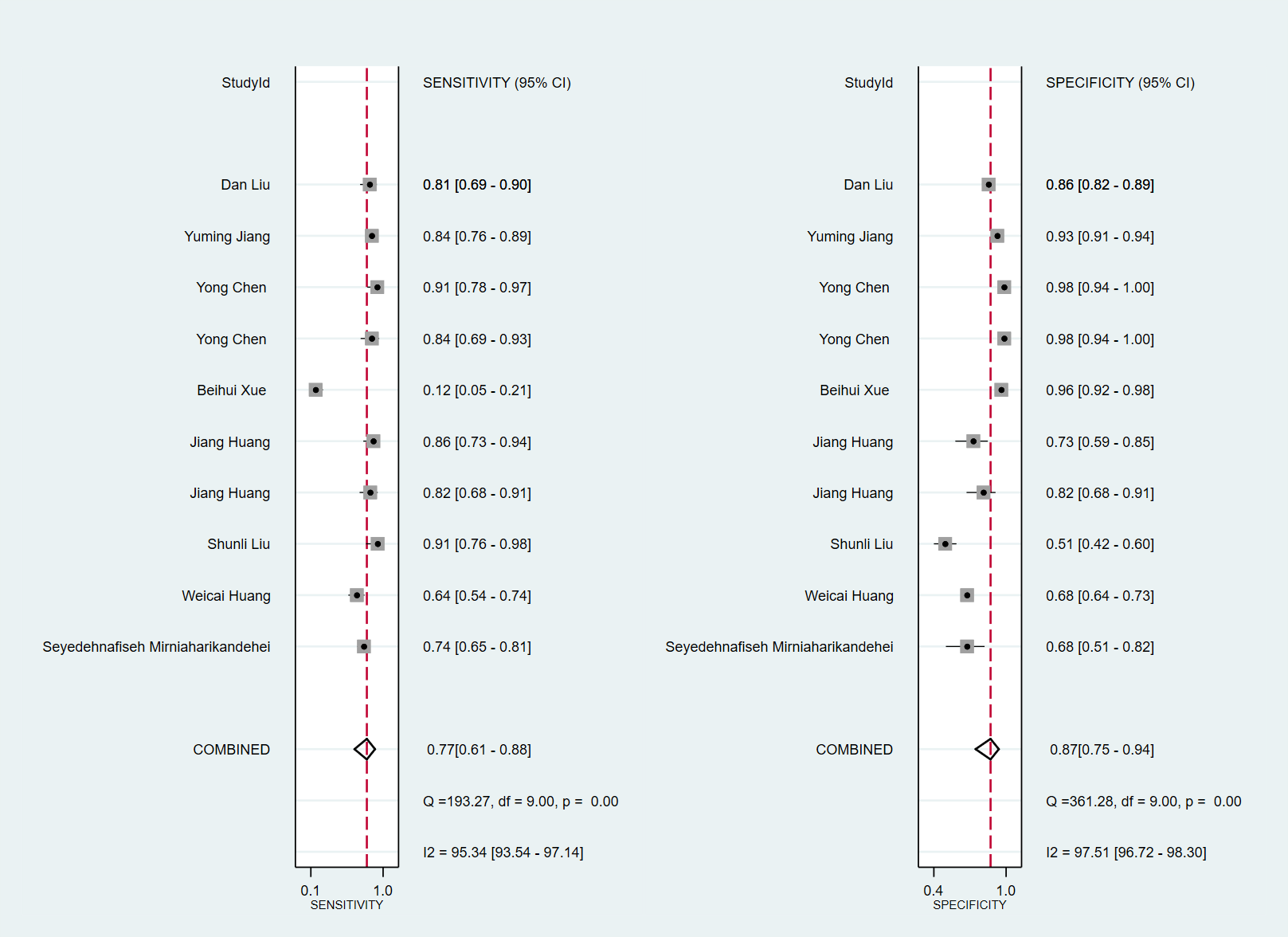


Fig. S2 Radiomics-based machine learning models in the training set


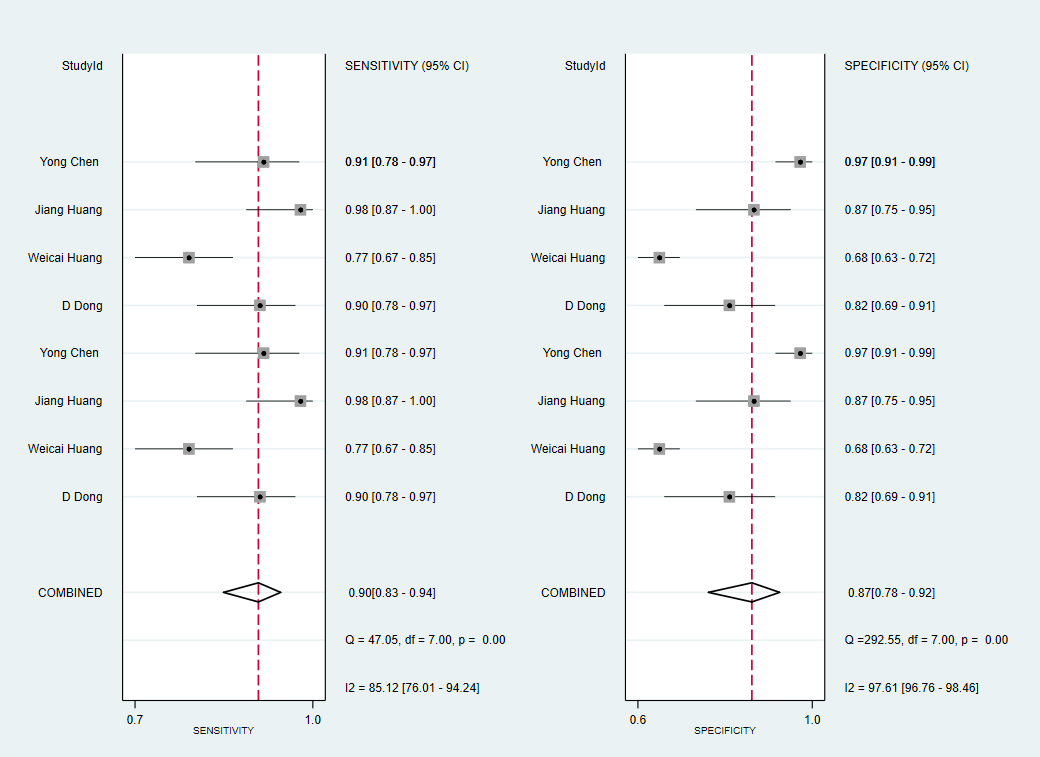


Fig. S3 Clinical features-based machine learning models in the training set


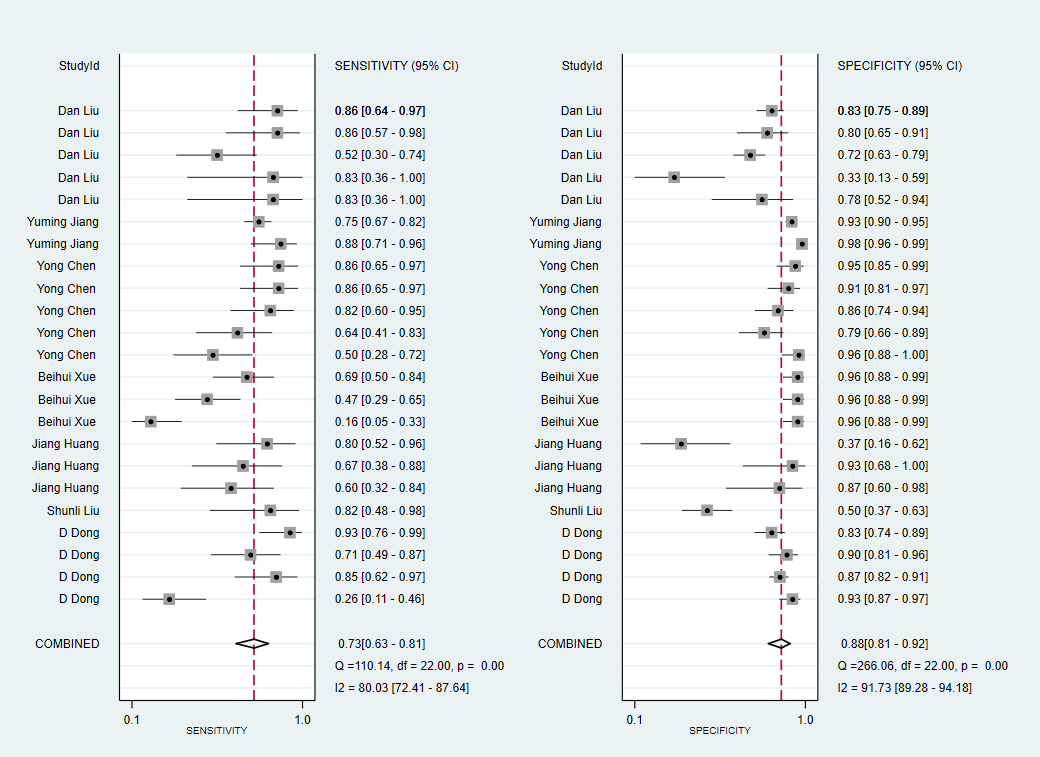


Fig. S4 Machine learning models based on both clinical features and radiomics in the training set


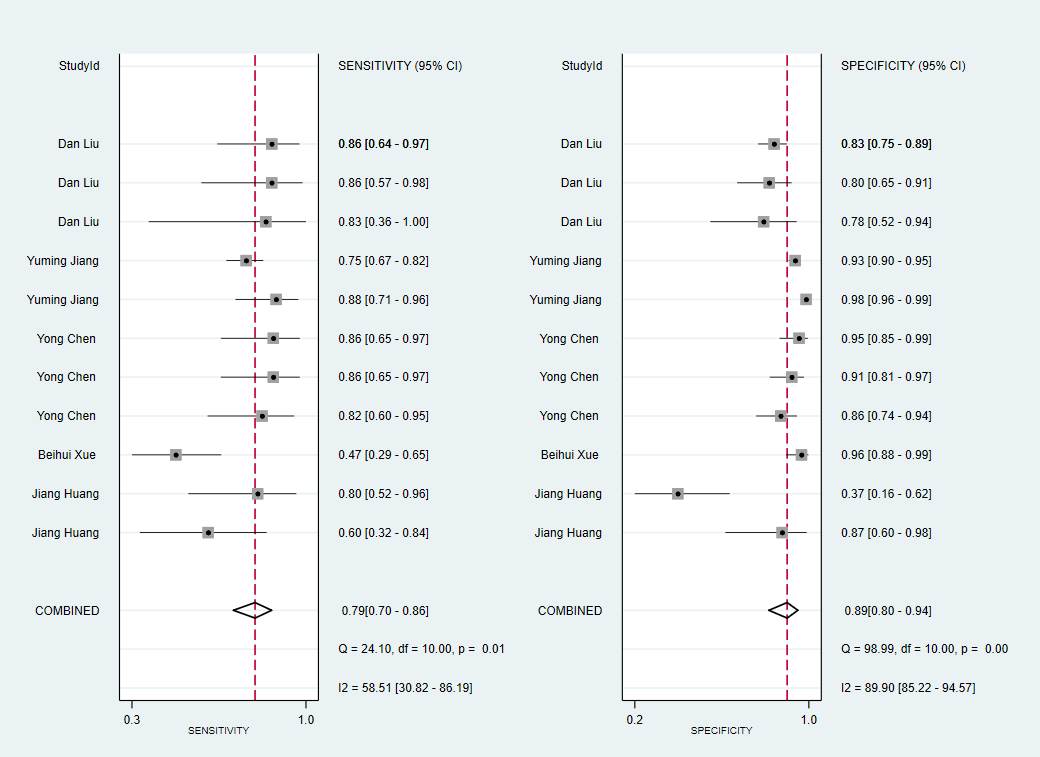


Fig. S5 Machine learning models in the validation set


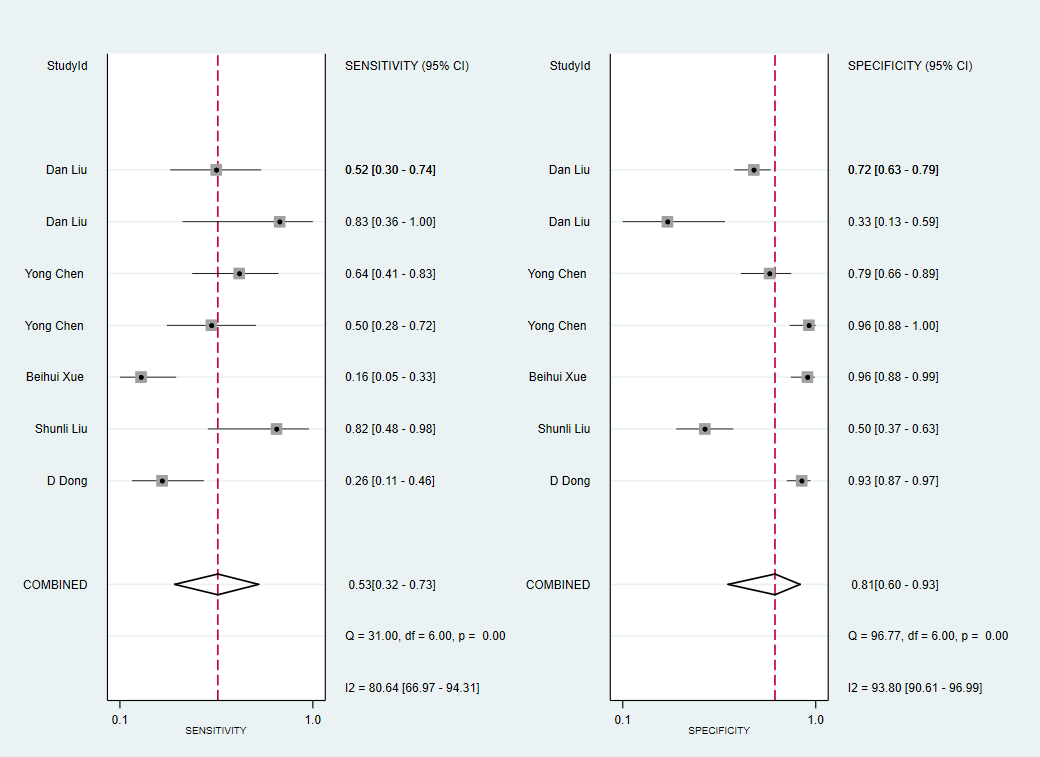


Fig. S6 Radiomics-based machine learning models in the validation set


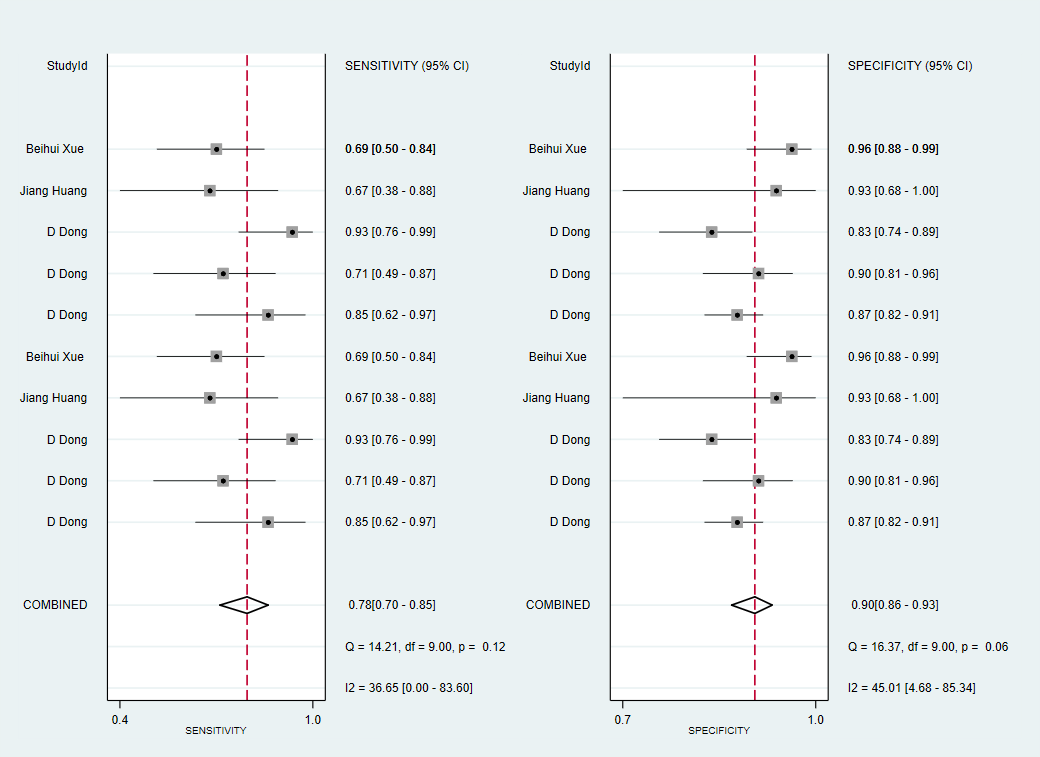


Fig. S7 Clinical features-based machine learning models in the validation set


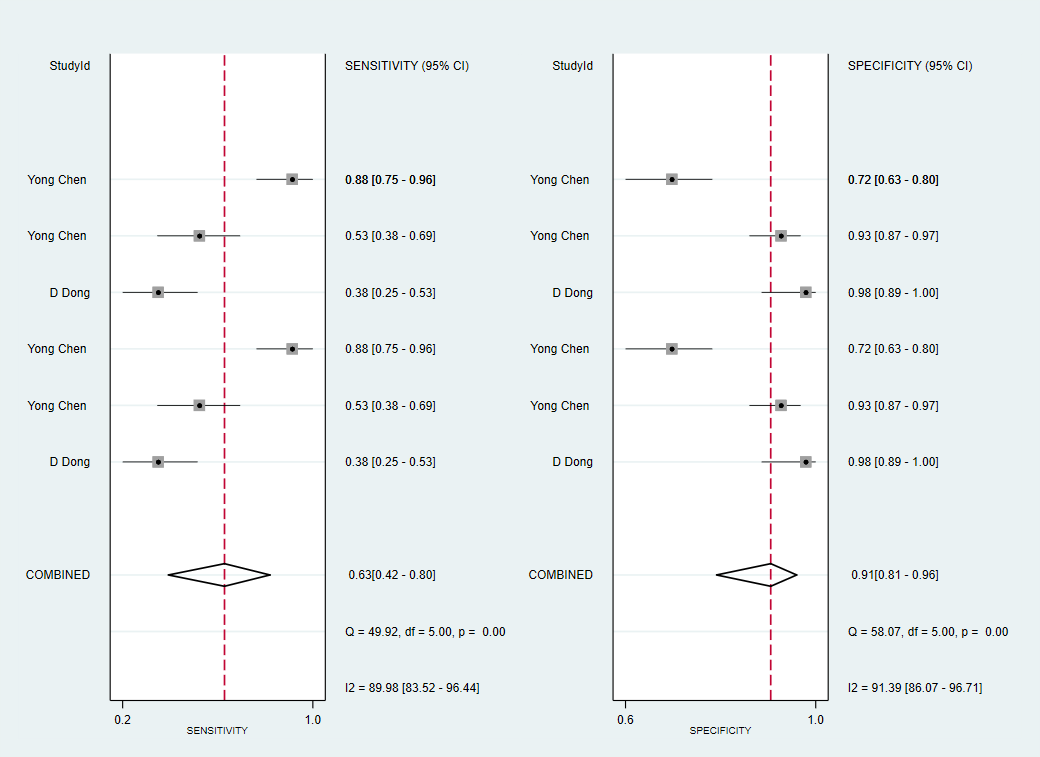


Fig. S8 Machine learning models based on both clinical features and radiomics in the validation set
